# Are Lewy Body Disease and Attention‐Deficit/Hyperactivity Disorder linked?

**DOI:** 10.1002/alz70857_103431

**Published:** 2025-12-25

**Authors:** Sara Becker, Baeleigh VanderZwaag, Hawra Al‐Khaz'Alya, Alexandra Wall, Brandy L. Callahan

**Affiliations:** ^1^ University of Calgary, Calgary, AB, Canada; ^2^ Hotchkiss Brain Institute, Calgary, AB, Canada; ^3^ University of Victoria, Victoria, BC, Canada; ^4^ Institute for Aging and Lifelong Health, Victoria, BC, Canada

## Abstract

**Background:**

Recent research suggests that, relative to controls, adults with attention‐deficit/hyperactivity disorder (ADHD) are more often diagnosed with Lewy Body Disease (LBD) in old age and people with LBD are more likely to report features of ADHD earlier in life. We examined the prevalence of childhood ADHD symptoms in older adults with Parkinson's disease (PD) and known early LBD markers in older adults with ADHD.

**Method:**

Participants aged 40+ were recruited from the local community in Calgary, Alberta and the Calgary Parkinson Research Initiative Registry. The following measures were administered: Montreal Cognitive Assessment, Barkley Adult ADHD Rating Scale‐IV Childhood symptoms, Beck Depression Inventory (BDI), a 5‐item self‐report questionnaire for autonomic dysfunction, Innsbruck REM Sleep Behavior Disorder (RBD) Inventory, and University of Pennsylvania Smell Identification Test. Kruskal‐Wallis *H* or Chi‐square tests analyzed group differences.

**Result:**

The sample consisted of ADHD (*n* = 40), PD (*n* = 35), and control (*n* = 37) participants and was largely female (61.6%). Mean age of the sample was 64.34 years (SD=11.03). There was no difference in cognition between groups, *H*(2,108)=4.42, *p* = .11. The ADHD group had significantly worse childhood ADHD symptoms than PD and control groups on inattention, hyperactive‐impulsive, and total ADHD symptom percentiles, *H*(2,111)>38.28, all *p* < 0.001). The ADHD group also had significantly more depressive symptoms than control participants (*F*(3,103)=8.34, *p* = .001). People with PD (44.1%) reported more problems with salivation than ADHD (30.8%) and control (10.8%) groups (*χ* ^2^(2,110)=9.91, *p* = .007), and more issues with constipation (67.6%) than ADHD (46.2%) and control (13.5%) groups (*χ* ^2^(2,110)=21.81, *p* < .001). Based on the RBD inventory cut‐off (≥0.25), 22.9% of ADHD, 53.1% of PD, and 12.5% of controls were classified as having probable RBD (*χ* ^2^(2,112)=13.82, *p* < .001). Participants with PD had worse olfaction than ADHD and control groups (*H*(2,112)=25.44, *p* < .001); 86% of participants with PD had moderate microsmia or worse, compared to 7.5% and 30% in the ADHD and control groups, respectively.

**Conclusion:**

Our data did not show a higher prevalence of childhood ADHD symptoms in people with PD, or that people with ADHD had increased LB markers. Our findings do not suggest that ADHD is an early stage or risk factor for LBD and there is no evidence that they are pathophysiologically linked.

